# A likely pathogenic *POLD1* variant associated with mandibular hypoplasia, deafness, progeroid features, and lipodystrophy syndrome in a Chinese patient

**DOI:** 10.1186/s12920-022-01374-x

**Published:** 2022-10-21

**Authors:** Bin Zuo, Hongen Xu, Zhaoyu Pan, Lu Mao, Haifeng Feng, Beiping Zeng, Wenxue Tang, Wei Lu

**Affiliations:** 1 Department of Otorhinolaryngology, The First Affiliated Hospital of Zhengzhou University, No. 1 Jian-she Road, Zhengzhou, 450052 China; 2 Precision Medicine Center, Academy of Medical Science, Zhengzhou University, No. 40 Daxuebei Road, Zhengzhou, 450052 China; 3 The Research and Application Center of Precision Medicine, The Second Affiliated Hospital of Zhengzhou University, No. 2 Jing-ba Road, Zhengzhou, 450014 China; 4 Henan Institute of Medical and Pharmaceutical Sciences, Zhengzhou University, No. 40 Daxuebei Road, Zhengzhou, 450052 China

**Keywords:** Mandibular hypoplasia, progeroid features, and lipodystrophy syndrome, MDPL, Sensorineural hearing loss, *POLD1*

## Abstract

**Background:**

Mandibular hypoplasia, deafness, progeroid features, and lipodystrophy syndrome (MDPL; OMIM# 615381) is a rare autosomal dominant disorder, with only a few reported cases worldwide. Herein, we describe the clinical features and underlying molecular etiology of MDPL syndrome in an 8-year-old Chinese patient.

**Methods:**

We performed otological, endocrine, ultrasound, and radiological examinations, as well as genetic testing. Additionally, the literature concerning MDPL was reviewed to do a retrospective analysis of the pathogenesis, genotype–phenotype correlation, and clinical management.

**Results:**

The proband was diagnosed with MDPL, presenting with mandibular hypoplasia, a characteristic facial appearance, lipodystrophy, and sensorineural hearing loss (SNHL). Whole-exome sequencing and bioinformatics analysis revealed a de novo missense variant in the *POLD1* gene, NM_002691.4:c.3185A>G (NP_002682.2:p.(Gln1062Arg)). The retrospective analysis showed wide variation in the MDPL phenotype, but the most frequent features included mandibular hypoplasia, characteristic facial appearance, lipodystrophy, and SNHL.

**Conclusions:**

This study supplements the mutational spectrum of *POLD1*. The genetic analysis contributes to the diagnosis of syndromic deafness, and it has a vital role in clinical management and future genetic consultation.

## Introduction

Mandibular hypoplasia, deafness, progeroid features, and lipodystrophy syndrome (MDPL; OMIM# 615381) is a rare disease characterized by mandibular hypoplasia, a characteristic facial appearance, lipodystrophy and sensorineural hearing loss (SNHL). Shastry et al. [1] first described MDPL in seven unrelated patients from mandibuloacral dysplasia (MAD) patients without pathogenic variants in known causal genes. In four unrelated patients with MDPL, Weedon et al. [2] first reported a recurrent heterozygous in-frame deletion of the *POLD1* gene, c.1812_1814del (p.(Ser605del)) through exome sequencing. The clinical manifestations of MDPL also include prominent eyes, crowded teeth, poor breast development in females, hepatomegaly, a small mouth, hepatic steatosis, cryptorchidism and hypogonadism in males, osteoporosis, joint contractures, kyphosis, scoliosis, a beaked nose, tight skin, scleroderma-like changes, telangiectases, skin atrophy, a high-pitched voice, insulin resistance, diabetes mellitus, abnormal liver function tests, and hypertriglyceridemia. MDPL should be differentiated from MAD (OMIM# 248370, 608612) and Werner syndrome (WS; OMIM# 277700). MAD was transmitted in an autosomal recessive pattern, and the etiology was attributed to *LMNA* or *ZMPSTE24* alterations. MAD patients presented mainly with growth retardation, craniofacial anomalies, skeletal anomalies, and skin changes. Compared to MAD patients, MDPL patients have a better overall prognosis, normal hair, no alopecia, clavicle dysplasia, or acroosteolysis [1]. WS, which results from pathogenic variants in the *WRN* gene, is an autosomal recessive disorder characterized by premature aging and an increased risk of various forms of cancer. Compared to WS patients, MDPL patients develop a facial appearance characteristic of premature aging at a younger age, but do not exhibit premature loss or graying of hair [3].

The present study reports an MDPL case in a Chinese family. Whole-exome sequencing identified a likely pathogenic variant in the *POLD1* gene. A literature review was performed to examine the phenotypic variability of MDPL.

## Materials and methods

### Patients and clinical investigations

An 8-year-old girl who suffered from hearing loss presented to the Department of Otorhinolaryngology, Affiliated Hospital of Zhengzhou University, China. Audiological evaluation revealed severe bilateral SNHL, with no indication of a conductive hearing loss. Data related to the pedigree of the patient were collected from her parents. The clinical assessment was performed, including audiological, endocrine, ultrasound, and radiological examinations. The project was approved by the Ethics Committee of the Affiliated Hospital of Zhengzhou University (No.: 2018008) and all described procedures were complied with the Helsinki Declaration. A signed informed consent form was obtained from the proband’s parents.

### Genetic testing and bioinformatics analysis

Whole blood (3 ml) was collected from the affected proband, her parents, and her younger sister. Genomic DNA was isolated from the blood following the manufacturer’s protocol of the Genomic DNA Purification kit (GenMagBio, Changzhou, China). The whole-exome sequencing procedures, including fragmentation, end-repair of genomic DNA, targeted enrichment, and sequencing, were identical to those described in previous studies [4]. After trimming the adapters and low-quality reads, cleaned reads were mapped to the human reference genome (version GRCh37). Bioinformatics analyses, including variant calling, annotation, and filtering, were performed as those described previously [4]. The ACMG (American College of Medical Genetics) standards and guidelines were used to interpret sequence variants [5]. Variant nomenclature was based on *POLD1* canonical transcript NM_002691.4. To verify the candidate variants and test for co-segregation in the kindred, Primer-BLAST was used to design primers for c.3185A>G (forward primer 5′-CAGGAGCCGTGTGTGAGTT-3′ and reverse primer 5′-TCACAGCTGGAAGGGGATG-3′). Then PCR and Sanger sequencing were carried out.

### Literature review

Using MDPL and *POLD1* as keywords, PubMed was searched for articles published between 2010 and 2021 on the genotype and phenotype of MDPL.

## Results

### Case presentation

**Fig. 1:**
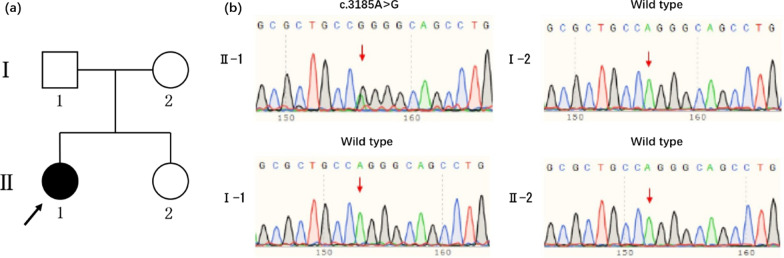
Pedigree and Sanger sequencing of the MDPL family. **a** Pedigree of the MDPL family. **b** Sanger sequencing

**Fig. 2:**
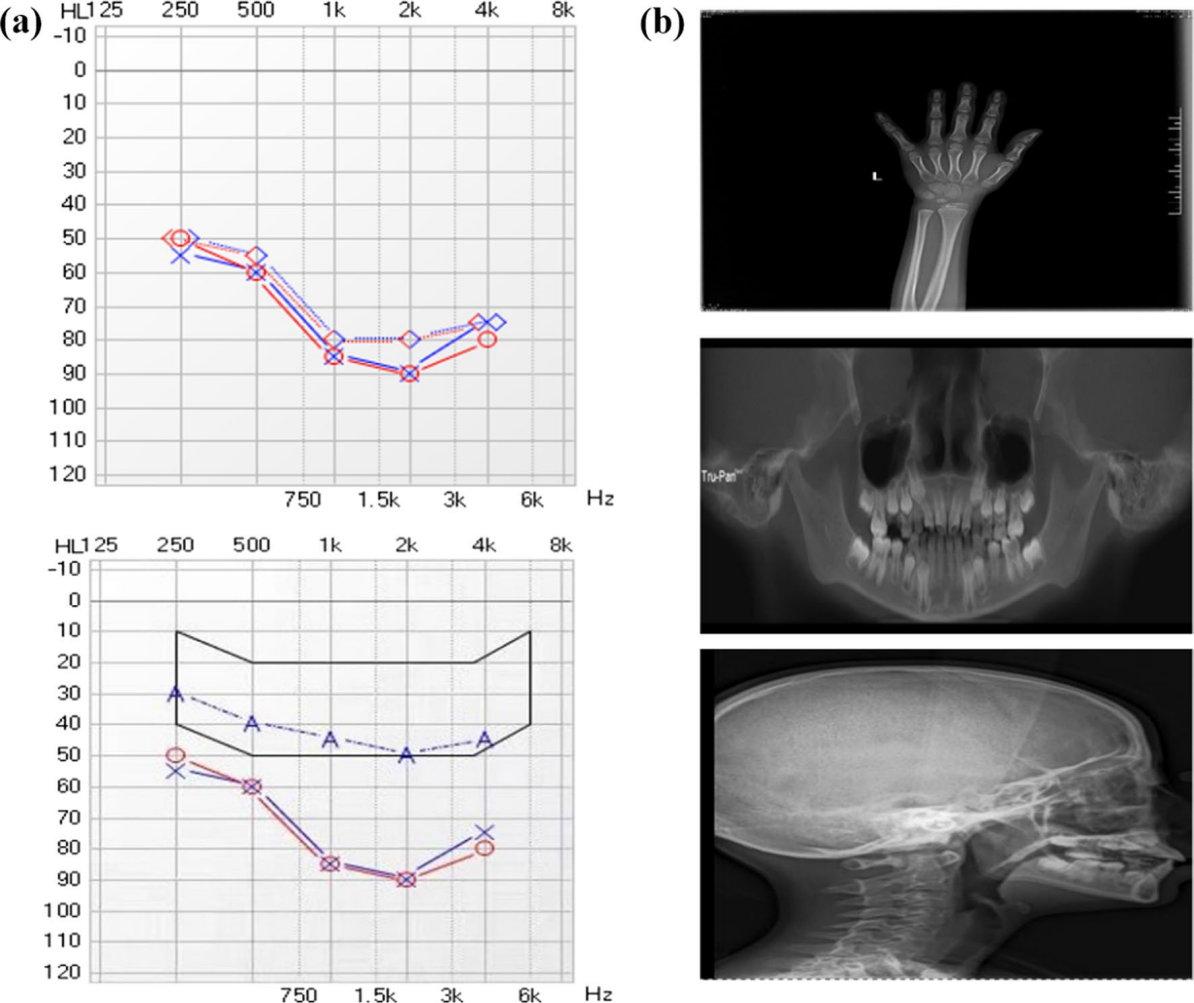
Facial phenotype, audiogram, and X-ray of the proband. **a** Bilateral severe sensorineural hearing loss revealed by pure-tone audiometry showed. The use of hearing aids led to a successful outcome. **b** When she was 6 years old, the bone age was only 3 years. X-ray showing crowded teeth and mandibular hypoplasia

The proband (II-1, Fig. 1a), aged 8-year-old, from a non-consanguineous Chinese parent. She was born at 32 weeks of gestation with a birth weight of 2.1 kg. She exhibited poor growth and thin limbs since the age of 4 years. She is allergic to fish, eggs, milk, and wheat. She had prominent eyes, a beaked nose, a small mouth, kyphosis, tight skin, and a high-pitched voice. She was diagnosed with severe bilateral SNHL on the basis of pure-tone audiometry tested at six years of age. Her bone age was delayed, and was estimated at three years (Fig. 2a, b). Distortion product otoacoustic emissions were absent at all frequencies on both sides. The auditory brainstem response was evaluated to determine the average (right, 100 dB nHL normal hearing level; left, 90 dB nHL) and bone conduction (right, 50 dB nHL; left, 50 dB nHL) thresholds. The auditory steady-state response thresholds at 500, 1,000, 2,000, and 4000 Hz were 70, 90, 90, and 90 dB nHL in the left ear, and were 70, 90, 90, and 80 dB nHL in the right ear, respectively. X-rays showed crowded teeth and mandibular hypoplasia (Fig. 2b). Temporal bone CT was normal. Osteoporosis, joint contractures, and insulin resistance were not observed. The levels of glucose, triglycerides, insulin, aspartate transaminase, alanine transaminase, and total bilirubin were within the normal limits. No internal organ abnormalities were observed by ultrasound. At the age of 8 years, the patient’s height and weight were 1.26 m and 16 kg, respectively. The follicle-stimulating hormone, luteinizing hormone, estradiol, and prolactin levels were 25.7 mIU/mL, 0.92 mIU/mL, 8.44 pg/mL, and 41.2 ng/mL, respectively. She had a normal intelligence level. The use of hearing aids in both ears provided good results. The proband's family members were unaffected and had normal hearing (Fig. 1a).

### Molecular diagnosis

The proband carried a missense variant of the *POLD1* gene, identified by whole-exome sequencing: c.3185A>G (Fig. 1b). The variant was validated in the family members using Sanger sequencing; the results showed that the proband’s parents and younger sister did not carry this variant, suggesting a de novo status in the proband (both maternity and paternity confirmed, PS2). The variant was not found previously in public databases (PM2_Supporting). The proband’s phenotype is highly specific for MDPL with a single genetic etiology (PP4). Multiple lines of computational evidence, including SIFT, Polyphen2, and MutationTaster, support its deleterious effect on the POLD1 protein (PP3). This variant has been submitted to the Clinvar database (SCV001477293) and classified as variant of uncertain significance (VUS). In summary, the variant c.3185A>G was reclassified as likely pathogenic.

## Discussions

### Mechanism

MDPL is caused by pathogenic variants in the *POLD1* gene, which encodes for the catalytic subunit (p125) of DNA polymerase delta (Polδ). The catalytic subunit is responsible for synthesizing the lagging strand DNA during DNA replication with both 5′- to 3′-polymerase activity and 3′- to 5′-exonuclease activity [6, 7]. Polδ is involved in DNA replication and maintains genomic stability. Downregulation of the p125 subunit causes genomic instability and ultimately DNA replication errors. The proofreading ability of Polδ results from its exonuclease activity, which is essential to ensure replication fidelity [6]. The molecular mechanism is still unclear. The p125 mutation leads to reduced genomic stability, cellular senescence, and apoptosis, which may cause deafness and progeroid features in MDPL patients.

### Molecular mapping

Encoded by the *POLD1* gene with a size of 34 kb and 29 exons, POLD1 has polymerase and exonuclease activities on its p125 catalytic subunit. POLD1 consists of putative nuclear localization signal (amino acids 4–19), exonuclease domain (amino acids 306–519), polymerase active site (amino acids 581–910), and ZnF domain (amino acids 1012–1083), which contains two conserved cysteine-rich metal-binding motifs: CysA and CysB [7]. The six previously reported *POLD1* pathogenic variants in MDPL patients are summarized in Table 1. Most variants were de novo. The hotspot of *POLD1* variants associated with MDPL is the c.1812_1814del. Weedon et al. [2] showed using in vitro functional studies that the c.1812_1814del resulted in inefficient interaction of DNA polymerase with dNTPs and incorporation into the extended DNA strand. However, the nucleic acid exonuclease activity was retained, implying that the proofreading function was normal and did not increase the rate of base substitution errors, resulting only in abnormal DNA synthesis. In our study, the missense variant c.3185A>G was found on the CysB motif (amino acids 1058–1076).

**Table 1:** *POLD1* pathogenic variants in MDPL

Nucleotide changes	Amino acid changes	Type of variants	Publications
c.[589_589+1del; 3298G>A]^a^	p.(/; Gly1100Arg)	/; Missense	Oh et al. [8]
c.1519C>T	p.(Arg507Cys)	Missense	Pelosini et al. [9] Lessel et al. [3]
c.1812_1814del	p.(Ser605del)	In-Frame	Weedon et al. [2] Lessel et al. [3] Reinier et al. [10] Elouej et al. [7] Okada et al. [11] Wang et al. [12] Fiorillo et al. [13] Sasaki et al. [14] Wang et al. [15] Murdocca et al. [6] Yu et al. [16] Zhou et al. [17]
c3185A> G	p.(Gln1062Arg)	Missense	This study
c.3199G>A	p.(Glu1067Lys)	Missense	Ajluni et al. [18]
c.3209T>A	p.(Ile1070Asn)	Missense	Elouej et al. [7]

Nucleotide numbering is based on GenBank reference sequence NM_002691.4

MDPL, mandibular hypoplasia, deafness, progeroid features, and lipodystrophy syndrome

^a^The first variant was originally described as “NM_001256849.1:c.584_585del” in the study

### Phenotypic variability

Lessel et al*.* [3] described eight *POLD1* pathogenic variants carriers who did not have hearing loss or mandibular hypoplasia; these patients had a lower incidence of metabolic abnormalities and joint contractures, indicating that *POLD1* pathogenic variants could result in a variable expression. The phenotypic variability of MDPL syndrome remains unclear. There are 33 reported cases of MDPL worldwide, with ~ 70% from Europe and the Americas and ~ 30% from Asia. Almost all reported cases of MDPL have mandibular hypoplasia, a characteristic facial appearance, lipodystrophy, and SNHL (Table 2). Additionally, all female patients have poor breast development. More than half of all patients have hepatic steatosis, joint contractures, kyphosis, skin changes, a high-pitched voice, and hypertriglyceridemia, with cryptorchidism and hypogonadism also seen in males. Lipodystrophy almost always occurs in early childhood, while hearing loss occurs in the first or second decade.

**Table 2:** A review of physical features of MDPL

Site	Features	Frequency	Shastry et al. [1], Spo N	Weedon et al.[2], Spo	Pelosini et al. [9], Spo	Reinier et al.[10], Spo	Lessel et al. [3]	Ajluni et al. [18]	Elouej et al. [7], Spo	Okada et al.[11], Spo	Wang et al. [12], Spo	Fiorillo et al.[13], Spo	Sasaki et al.[14], Spo	Wang et al. [15], Spo	Oh et al. [8]	Yu et al. [16], Spo	Zhou et al. [17], Spo	Murdocca et al.[6]	This study Proband
Weight	Normal birth weight	23/23	7/7	2/2	+	+	4/4	NA	2/2	+	+	NA	+	NA	+	+	+	NA	NA
Face	Mandibular hypoplasia	32/33	7/7	2/2	+	+	7/8	2/2	2/2	+	+	+	+	+	–	+	+	+	+
	Progeroid appearance	8/11	NA	NA	+	+	NA	0/2	NA	NA	+	NA	+	+	–	+	+	NA	+
Ears	Sensorineural hearing loss	26/32	7/7	2/2	+	+	5/8	0/2	1/2	+	+	+	+	+	+	+	+	NA	+
Eyes	Prominent eyes	14/16	7/7	NA	–	+	NA	NA	NA	+	+	NA	+	NA	–	+	+	NA	+
Nose	Beaked nose	29/30	7/7	2/2	+	+	8/8	NA	2/2	+	+	+	+	+	–	+	NA	+	+
Mouth	Small mouth	18/19	7/7	NA	+	+	NA	NA	NA	+	NA	+	+	+	–	+	NA	+	+
Teeth	Crowded teeth	19/28	2/7	2/2	+	+	5/8	NA	2/2	+	+	NA	NA	+	–	+	+	NA	+
Breasts	Poor breast development (female)	8/8	2/2	M	+	+	NA	NA	1/1	M	M	NA	NA	NA	M	+	+	+	NA
Liver	Hepatomegaly	6/14	3/5	0/2	+	+	NA	NA	0/2	NA	NA	NA	NA	NA	–	NA	+	NA	–
	Hepatic steatosis	12/20	2/5	1/1	+	+	1/2	NA	0/2	+	+	NA	+	+	–	+	NA	+	-
Internal genitalia	Cryptorchidism (male)	7/10	3/5	2/2	F	F	NA	NA	1/1	+	NA	F	F	F	-	F	F	F	F
Skeleton	Osteoporosis	9/24	1/7	2/2	NA	NA	2/7	NA	1/2	NA	+	–	NA	+	–	NA	NA	+	–
	Joint contractures	17/30	5/7	2/2	–	+	1/8	2/2	2/2	+	+	+	NA	NA	–	–	+	NA	-
Spine	Kyphosis	6/10	NA	2/2	+	–	NA	NA	1/2	–	NA	+	NA	NA	–	NA	NA	NA	+
	Scoliosis	4/9	NA	2/2	+	–	NA	NA	1/2	–	NA	NA	NA	NA	–	NA	NA	NA	–
Skin	Tight skin	18/26	3/7	2/2	–	–	8/8	NA	NA	+	+	NA	–	NA	–	+	+	NA	+
	Scleroderma-like changes	5/15	1/7	NA	+	+	NA	NA	NA	–	NA	NA	–	NA	–	+	NA	+	–
	Telangiectases	17/26	4/6	2/2	–	+	5/8	NA	1/2	+	NA	NA	+	NA	–	+	NA	+	–
	Skin atrophy	9/15	7/7	NA	–	+	NA	NA	NA	–	–	NA	+	NA	–	–	NA	NA	–
Soft tissues	Lipodystrophy	32/33	7/7	2/2	+	+	8/8	2/2	2/2	+	+	+	+	+	–	+	+	+	+
	Loss of subcutaneous fat, particularly affecting the limbs	23/31	3/7	NA	–	+	8/8	2/2	1/2	+	+	+	+	+	–	–	+	+	+
	Loss of subcutaneous fat, generalized	7/31	4/7	NA	+	–	0/8	0/2	1/2	–	–	–	–	–	–	+	–	–	–
	Increased visceral fat	14/22	2/5	2/2	+	+	1/2	NA	0/2	+	+	+	+	+	–	+	NA	+	–
Voice	High-pitched voice	13/25	1/7	2/2	NA	+	5/8	NA	0/2	+	+	NA	+	NA	–	NA	NA	NA	+
Endocrine	Insulin resistance	11/22	1/7	2/2	+	+	NA	2/2	NA	–	–	+	+	+	–	–	NA	+	–
	Diabetes mellitus	13/33	2/7	2/2	–	+	2/8	2/2	0/2	+	–	–	+	–	–	–	+	+	–
	Hypogonadism (male)	5/8	NA	2/2	F	F	1/2	NA	0/1	+	+	F	F	F	–	F	F	F	F
Others	Abnormal liver function tests	7/15	NA	2/2	NA	+	0/3	2/2	0/1	+	–	NA	+	NA	–	–	NA	NA	–
	Hypertriglyceridemia	20/29	6/7	2/2	+	+	5/6	2/2	0/2	–	+	NA	+	–	–	–	+	NA	–
	Onset of lipodystrophy in early childhood	24/25	5/5	2/2	+	+	4/4	NA	2/2	+	+	+	+	+	–	+	+	+	+
	Onset of hearing loss in first or second decade	23/28	7/7	1/2	+	+	5/8	NA	1/1	+	+	+	+	+	NA	–	+	NA	+

MDPL, mandibular hypoplasia, deafness, progeroid features, and lipodystrophy syndrome; N, none of molecular genetic diagnosis; Spo, Sporadic; F, female; M, male; NA, not available

### Treatment and prevention

Audiological, ophthalmological, endocrine, ultrasound, and radiological examinations should be regularly performed for MDPL patients. Additionally, levels of glucose, triglycerides, insulin, and liver enzymes should be measured. SNHL is usually bilateral and sometimes progressive, with onset in the first or second decade of life. The most common intervention for SNHL is a hearing aid (Table 3). The proband in our study presented with severe SNHL and had a successful outcome with the use of a hearing aid. Cochlear implants can be used for patients with profound deafness who have poor outcomes with hearing aids. However, the effectiveness of cochlear implants in MDPL patients remains to be studied.

**Table 3:** Interventions of SNHL in reported MDPL

Previous publications	Age at SNHL onset (years)	Interventions
Shastry et al. [1], patient 100.3	18	NA
Shastry et al. [1], patient 200.5	15	NA
Shastry et al. [1], patient 300.4	7	NA
Shastry et al.[1], patient 400.3	8	NA
Shastry et al. [1], patient 500.4	16	NA
Shastry et al. [1], patient 600.4	14	NA
Shastry et al. [1], patient 700.3	6	NA
Weedon et al. [2], patient 1	12	Bilateral hearing aids
Weedon et al. [17], patient 2	33	NA
Pelosini et al. [9]	25	Hearing aid
Reinier et al. [10]	10	NA
Lessel et al. [3], patient 1	N	NA
Lessel et al. [3], patient 2	12	NA
Lessel et al. [3], patient 3	11	NA
Lessel et al. [3], patient 4	N	NA
Lessel et al. [3], patient 5	14	NA
Lessel et al. [3], patient 6	14	NA
Lessel et al. [3], patient 7	N	NA
Lessel et al. [3], patient 8	NA	NA
Ajluni et al. [18], patient 6	N	N
Ajluni et al. [18], patient 7	N	N
Elouej et al. [7], patient 1	N	N
Elouej et al. [7], patient 2	4	Bilateral hearing aids
Okada et al. [11]	7	Hearing aids
Wang et al. [12]	10	Hearing aids
Fiorillo et al. [13]	2	NA
Sasaki et al. [14]	Puberty	NA
Wang et al. [15]	15	NA
Oh et al. [8], SB127-219	NA	NA
Oh et al. [8], SB127-277	NA	NA
Yu et al. [16]	25	NA
Zhou et al. [17]	20	NA
Murdocca et al. [6]	NA	NA
This study proband	6	Bilateral hearing aids

SNHL, sensorineural hearing loss; MDPL, mandibular hypoplasia, deafness, progeroid features, and lipodystrophy syndrome; N, no; NA, not available

MDPL is an autosomal dominant trait that has a 50% probability of being inherited by the offspring. Genetic counseling should be recommended for the proband and her future spouse. A preimplantation genetic diagnosis may help prevent MDPL in the offspring. Whole-exome sequencing contributes to MDPL diagnosis and is beneficial for genetic counseling of MDPL patients.

## Data Availability

All data supporting the findings of this study are available on request from the corresponding author. The likely pathogenic variant have been submitted to ClinVar (https://www.ncbi.nlm.nih.gov/clinvar/) with the accession number (SCV002546239).
